# Early Screening for Long QT Syndrome and Cardiac Anomalies in Infants: A Comprehensive Study

**DOI:** 10.3390/clinpract14030082

**Published:** 2024-05-31

**Authors:** Luana Nosetti, Marco Zaffanello, Carolina Lombardi, Alessandra Gerosa, Giorgio Piacentini, Michele Abramo, Massimo Agosti

**Affiliations:** 1 Pediatric Sleep Disorders Center, Division of Pediatrics, “F. Del Ponte” Hospital, University of Insubria, 21100 Varese, Italy; luana.nosetti@uninsubria.it (L.N.);; 2 Department of Surgery, Dentistry, Pediatrics and Gynecology, University of Verona, 37100 Verona, Italy; 3 Sleep Disorders Center, Department of Cardiology Istituto Auxologico, IRCCS, 20149 Milan, Italy; 4 Department of Medicine and Surgery, University of Milano-Bicocca, 20126 Milan, Italy; 5 Woman and Child Department, Varese Hospital, Insubria University, Via Ravasi 2, 21100 Varese, Italy; massimo.agosti@uninsubria.it

**Keywords:** children, congenital heart disease, electrocardiogram, infant, long QTc syndrome, neonatal screening, sudden infant death syndrome

## Abstract

(1) Background: Sudden Infant Death Syndrome (SIDS) represents sudden and unexplained deaths during the sleep of infants under one year of age, despite thorough investigation. Screening for a prolonged QTc interval, a marker for Long QT Syndrome (LQTS), should be conducted on all newborns to reduce the incidence of SIDS. Neonatal electrocardiograms (ECGs) could identify congenital heart defects (CHDs) early, especially those not detected at birth. Infants with prolonged QTc intervals typically undergo genetic analysis for Long QT Syndrome. (2) Methods: The study involved infants aged 20–40 days, born with no apparent clinical signs of heart disease, with initial ECG screening. Infants with prenatal diagnoses or signs/symptoms of CHDs identified immediately after birth, as well as infants who had previously had an ECG or echocardiogram for other medical reasons, were excluded from the study. We used statistical software (SPSS version 22.0) to analyze the data. (3) Results: Of the 42,200 infants involved, 2245 were enrolled, with 39.9% being males. Following this initial screening, 164 children (37.8% males) with prolonged QTc intervals underwent further evaluation. Out of these 164 children, 27 children were confirmed to have LQTS. However, only 18 children were finally investigated for genetic mutations, and mutations were identified in 11 tests. The most common mutations were *LQT1* (54.5%), *LQT2* (36.4%), and *LQT3* (1 patient). Treatment options included propranolol (39.8%), nadolol (22.2%), inderal (11.1%), metoprolol (11.1%), and no treatment (16.7%). The most common abnormalities were focal right bundle branch block (54.5%), left axis deviation (9.2%), and nonspecific ventricular repolarization abnormalities (7.1%). Multiple anomalies were found in 0.47% of children with focal right bundle branch block. Structural abnormalities were associated with specific features in 267 patients (11.9%), primarily isolated patent foramen ovale (PFO) at 61.4%. (4) Conclusions: This screening approach has demonstrated effectiveness in the early identification of LQTS and other cardiac rhythm anomalies, with additional identification of mutations and/or prolonged QTc intervals in family members. Identifying other ECG abnormalities and congenital heart malformations further enhances the benefits of the screening.

## 1. Introduction

The term SIDS (Sudden Infant Death Syndrome) refers to the seemingly unexplained and sudden death during sleep of an infant under one year old after a thorough investigation [1,2]. In 1995, the syndrome was documented at 5.6 cases per 10,000 births [3], varying between 2 and 5 per 10,000 live births in most countries [4]. A noteworthy observation is that fifty percent of deaths occur between 7.6 and 17.6 weeks from birth [2,3]. The causes are believed to be multifactorial, involving various internal and external factors, including sleeping position [5,6,7] and exposure to cigarette smoke [8,9]. Among the various protective factors are breastfeeding [10] and pacifier use [11].

Furthermore, endogenous causes may encompass systemic anomalies, neurological or autonomic dysfunctions [12], prematurity [3], and pregnancy-related factors [13]. In numerous SIDS cases, immune system activation [6] and down-regulation of the anti-inflammatory pathway have been noted, rendering the infant more susceptible to infections [14]. Metabolic disorders appear to account for 2% of SIDS cases [6,15].

Neonatal arrhythmias, categorized as either benign or non-benign, include supraventricular tachycardia (SVT), ventricular tachycardia (VT), abnormalities in atrioventricular (AV) conduction, and genetic arrhythmias like congenital Long QT Syndrome (LQTS) [16]. Specific genes have been linked to the development of arrhythmias in SIDS cases [17]. Postmortem genetic screening on cardiac arrhythmia-related genes aids in identifying the cause of death and identifying at-risk family members [18,19].

A molecular association between LQTS and SIDS has been documented [20]. Infants who succumbed to SIDS exhibit a prolonged corrected QT interval (QTc) compared to survivors and infants who died from other causes [21,22]. In 9.5% of SIDS cases, functionally significant genetic variants in LQTS genes were identified [23]. However, there is currently no comprehensive genetic test to identify infants at risk of SIDS, and the field is still grappling with methodological challenges [24,25,26].

Newborns with genetic arrhythmias, including ventricular tachycardia (VT), fibrillation, Long QT Syndrome (LQTS), or high-grade atrioventricular (AV) block, might be at an increased risk for congenital heart malformations (CHDs) [27], which are the most commonly diagnosed genetic disorders in newborns. In Europe, the reported prevalence of CHDs at birth is 8.2 cases per 1000 live births [28]. Over the past three decades, the incidence of CHDs has remained stable, suggesting limited progress in prevention strategies [29].

A neonatal arrhythmogenic cardiomyopathy (ACM) should be considered in the differential diagnosis of LQTS in cases of SIDS. ACM is often linked to sudden cardiac arrest, affecting approximately 1 in 2000 to 1 in 5000 individuals [30]. Furthermore, molecular screening was performed on seven LQTS-associated genes (KCNQ1, KCNH2, SCN5A, KCNE1, KCNE2, KCNJ2, and CAV3). The study showed that sudden arrhythmic death is an essential contributor to SIDS [23]. A literature review mentions that genetic testing plays an essential role in diagnosing LQTS. At least 15 genes have been identified as playing a role in causing autosomal dominant congenital LQTS [31]. A later review reports that several of the minor LQTS-susceptibility genes, previously thought to be responsible for ~5–10% of non-syndromic LQTS cases, may be downgraded to the status of genes with limited or disputed evidence and, at most, be relegated to roles as oligogenic/polygenic contributors [32]. Another study [33] does not specifically mention a neonatal form of ACM in the context of LQTS or SIDS. However, in the neonatal context, it is important to distinguish between LQTS and ACM as they can present with similar symptoms, such as sudden cardiac arrest. Ultimately, the distinction between LQTS and ACM can be made based on genetic testing, ECG findings, family history, and imaging studies [34,35].

To reduce the incidence of SIDS, screening for LQTS should be sensitive enough and conducted for all newborns [36]. Additionally, the use of a neonatal electrocardiogram (ECG) could facilitate early detection of CHDs, especially those not apparent at birth [37,38].

Early ECG screening for LQTS and cardiac anomalies in infants is hindered by insufficient studies [34]. Large-scale studies with groups of participants (cohort studies) are crucial to fill these knowledge gaps. These studies will help identify the best way to screen infants and develop a cost-effective, thorough genetic test. This test would diagnose infants susceptible to SIDS, including those with LQTS and other heart conditions [34,39,40].

### Aims of the Study

This study looks back at medical records (retrospective) to assess how well newborn ECGs can spot children who might develop heart problems later in life (cardiovascular mortality and morbidity). This includes conditions like irregular heartbeats (arrhythmias), LQTS, and CHDs that have not been diagnosed yet.

## 2. Materials and Methods

### 2.1. Patients’ Enrolment

This retrospective study (covering the period from 2001 to 2017) was carried out at the Sleep-Related Respiratory Disorders clinic for ALTE/BRUE and SIDS at the University of Insubria and the Pediatric Cardiology Department of the Filippo Del Ponte Hospital in Varese, Italy.

**Figure 1:**
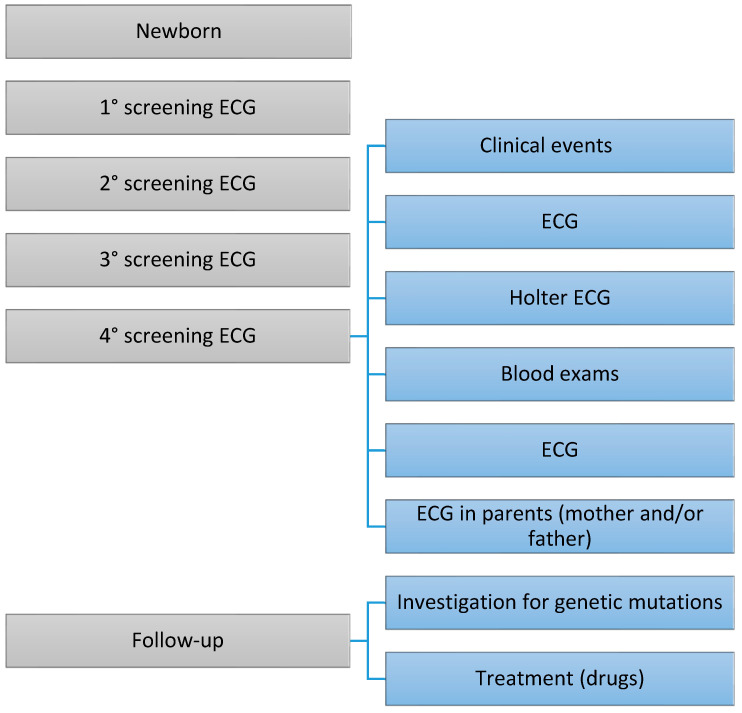
The figure depicts a series of phases that occur over time. The frequency of examinations may vary depending on the severity of the congenital heart defect (CHD). Similarly, the treatment for CHDs can differ based on the specific type of cardiac condition.

All healthy infants underwent initial routine ECG screening, primarily to investigate LQTS (Figure 1). Infants with prolonged QT intervals underwent follow-up and analysis for genetic mutations associated with LQTS. The study aimed to identify other CHDs that may have gone unrecognized at birth.

The study included infants between 20 and 40 days who were born clinically healthy and had undergone initial routine ECG screening. Infants with prenatal diagnoses or signs/symptoms of CHDs identified immediately after birth, as well as those who underwent an ECG or echocardiogram for other clinical or historical indications, were excluded from the study.

### 2.2. Electrocardiographic Neonatal Screening

Analyzing paper medical records enabled the identification of infants with a prolongation of the QTc interval observed in the first and/or subsequent ECG tracings taken at one- or two-week intervals. Twelve-lead ECGs were recorded on millimeter paper at 25 mm/sec speed using a Philips PageWriter TC50 electrocardiograph (Philips S.p.A., Viale Sarca 235, 20126 Milan, Italy). Pediatric cardiologists manually analyzed the ECG tracings, following the European Society Guidelines for interpreting neonatal ECGs [41]. QTc was considered prolonged when the QT interval, calculated with the Bazett formula, was ≥435 ms [42].

Children with prolonged QTc (≥435 ms) confirmed in multiple consecutive tracings (between three and four ECGs) were then subjected to a more in-depth screening (Figure 1).

### 2.3. Laboratory and Instrumental Tests

In some children, the following investigations were conducted to rule out the secondary causes of QTc interval prolongation (Figure 1): blood tests, Holter ECG, and 12-lead ECG on family members (mainly the mother and/or father). Additionally, selected children underwent an echocardiogram to identify any previously unrecognized CHDs. Children with persistent QTc prolongation were then evaluated for the potential development of symptoms related to LQTS, the results of the Holter ECG, and the possibility of a treatment plan.

### 2.4. Genetic Analysis

Next-generation sequencing (NGS) was used to identify pathogenic mutations in genes associated with LQTS and SIDS in the patients and their family members (*KCNQ1*, *KCNH2*, *SCN5A*, *KCNE1*, *KCNE2*, and *KCNH2*). No other genes were investigated at the time of the screening program. This approach focused on the significant LQTS-susceptibility genes. However, some minor susceptibility genes with limited or contested evidence could act as secondary contributors in a complex genetic picture (oligogenic/polygenic) [32].

### 2.5. Statistical Analysis

We recorded the data in a Microsoft^®^ Excel^®^ database and statistically analyzed them using SPSS version 22.0 (SPSS Inc., Chicago, IL, USA). The different categories within the data are shown by the number of times each category appears (n) and the percentage of observations that fall into each category (%).

Continuous variables are reported with mean, standard deviation (SD), and minimum (Min) and maximum values (Max). Group characteristics are expressed as mean, SD, standard error (SE), 95% confidence interval (95% CI), and Min and Max for the variables of interest. We employed the Mann–Whitney U test to determine if there were any statistically meaningful variations between the study groups.

### 2.6. Interpretation of the Results

After two authors (M.Z. and L.N.) interpreted the statistical analysis results, data tables were also analyzed using Google Gemini 1.0 Pro and Microsoft^®^ Copilot by OpenAI and Microsoft (2024) to explore alternative interpretations and identify consistencies. Discrepancies in the interpretations, particularly those arising from using Google Gemini 1.0 Pro and Microsoft^®^ Copilot, were discussed with a third author (M.P.) to ensure robust conclusions.

## 3. Results

### 3.1. Electrocardiographic Screening

The study recruited 42,200 infants, representing 82.4% of all children born between 2001 and 2017. Table 1 shows the proportion of children (with the percentage of males in brackets) across consecutive follow-up ECGs (ECG 1.0 to ECG 4.0) whose QTc values were at or above predefined cut-off values (listed in the first column).

At baseline (ECG 1.0), 2245 children were enrolled, which equates to 5.32% of those screened. Males comprised 39.9% of this group. For example, 37.8% of the subjects (with 37.6% being males) had a QTc ≥ 445 ms at the first follow-up ECG (ECG 1.0). This percentage increased to 57.7% (with 13.3% males) of enrolled infants with QTc ≥ 445 ms by the fourth follow-up ECG (ECG 4.0).

Table 1 also presents data for ECGs labeled ECG 1.1 to ECG 4.1. Only 164 subjects (representing 0.39% of those screened; 37.8% male) were enrolled at baseline (ECG 1.1). Similar to the previous table, the proportion of subjects with prolonged QTc (≥445 ms) increased across follow-up ECGs (e.g., 53.0% at ECG 1.1 to 60.9% at ECG 4.1).

Finally, the last column of Table 1 shows QTc values from Holter ECGs—only 44 subjects (representing 1.04‰ of those screened; 31.8% male) were enrolled for this. A significant portion (95.5%; 31.0% male) had a QTc ≥ 445 ms, with 68.2% (26.7% male) exceeding 455 ms.

Table 2 shows the mean QTc value ± SD for infants with QTc values exceeding predefined cut-off values (listed in the first column) across consecutive follow-up ECGs (ECG 1.0 to ECG 4.0 and ECG 1.1 to ECG 4.1) and a Holter ECG (last column). For example, the mean QTc ± SD for children with QTc ≥ 445 ms is 455.2 ± 8.7 ms at ECG 1.0, increasing to 458.5 ± 9.0 ms by ECG 4.1. Similarly, the mean QTc ± SD from a Holter ECG for those with QTc ≥ 445 ms is 467.2 ± 18.3 ms.

### 3.2. Genetic Investigation of LQTS

Table 3 shows the mean and SD of QTc values from ECG 1.1 to ECG 4.1 and Holter ECG. Not all completed the follow-up; only 18 underwent genetic examination (representing 0.43‰ of those initially screened).

Among the 18 children examined genetically, mutations were identified in 11 (61.1%), with no mutations found in the remaining 7 children (Table 4). The other seven children spontaneously withdrew from the study and were lost to follow-up.

Table 5 presents the genetic mutations detected in 11 children. One girl carried a *SCN5A* mutation (*c.647C>T*). Three girls had mutations in the *KCNH2* gene (*LQT2*): one with a nucleotide substitution (*c.1196C>T*) and another with a different substitution (*c.3367G>C*). A single girl harbored nucleotide substitutions in both *KCNH2* (*c.2560T>G*) and *SCN5A* (*c.5845G>A*). Follow-up data for this girl were unavailable.

Follow-up assessments revealed that most patients were asymptomatic. However, one patient with a *KCNQ1* mutation and polymorphisms in other genes (*SCN5A*-*H558R*, *KCNH2*-*K897K*, and *KCNE1*-*S38G*) experienced a syncopal episode. Five children (three males and two siblings) had heterozygous mutations in *KCNQ1* (*LQT1*). Another male child had a known *LQT1* mutation in his family history (present in his mother and sister). Six children (including two siblings, three females and three males) reported a positive family history. Eight out of the eleven children received a therapeutic protocol. Data were incomplete for a female carrier of both *KCNH2 (c.2560T>G*) and *SCN5A* (*c.5845G>A*), as well as for a female carrier of *KCNQ1* (*LQT1*). Additionally, a female carrier of *KCNH2* (*c.1196C>T*) was not undergoing any therapy.

In summary, the *KCNQ1* (*LQT1*) mutation was present in 54.5% of patients, the *KCNH2* (*LQT2*) mutation in 36.4% of patients, and the *SCN5A* (*LQT3*) mutation in one patient.

Table 4 presents neonatal ECG screening results for Long QTc in seven children (six females and one male) where specific mutations were not identified. QTc values were measured through both standard ECG and Holter ECG, revealing variations between children (minimum 402 ms–maximum 494 ms; minimum 440 ms–maximum 537 ms for Holter ECG).

Prescribed therapy varied among patients, with beta-blockers such as propranolol, nadolol, and metoprolol being the primary medications used. Propranolol is administered at a dosage of 2 mg/kg, with frequencies ranging from 1 to 3 times daily, depending on the individual case. Nadolol is given thrice daily at a dosage of ¾ of a tablet (40 mg). Metoprolol is administered at a dosage of 8 mg twice a day.

In summary, the following drugs were used for the treatment of Long QTc: propranolol (50% of children), nadolol (22.2% of children), metoprolol (11.1% of children), with 16.7% of children receiving no treatment.

### 3.3. Single and Multiple ECG Abnormalities

Table 6 provides details on single ECG abnormalities other than prolonged QTc that were found during neonatal screening for Long QTc in 1805 children (4.3% of those screened). The most frequent abnormality was the focal right bundle branch block, affecting 54.5% of children in this group. Left axial deviation was present in 9.2% of children with single anomalies, followed by nonspecific ventricular repolarization abnormalities (7.1%), ventricular extrasystole (4.6%), supraventricular extrasystole (4.5%), and complete right bundle branch block (3.7%). These results suggest a variety of electrical heart anomalies in children at risk of Long QTc, with a significant prevalence of focal right bundle branch block.

Table 6 also presents information on multiple ECG abnormalities, other than pro-longed QTc, identified during neonatal screening for Long QTc in 198 children (0.47‰ of children screened). Out of all the combinations observed, the most common one was right ventricular hypertrophy along with focal right bundle branch block. This combination affected 61% of the children in this group. Focal right bundle branch block combined with left axis deviation was found in 26% of children with multiple abnormalities. Notably, two-thirds (66.7%) of children with multiple ECG anomalies had focal right bundle branch block in conjunction with other electrical abnormalities.

Table 7 details the frequency of structural heart abnormalities associated with ECG abnormalities found in 267 neonates screened for Long QTc (6.3‰ of the total screened; 11.9% of those enrolled at ECG 1.0). The most frequent abnormality was isolated PFO in 164 patients (61.4%). PFO was associated with atrial septal defect ostium secundum (ASD OS) in 25 (9.4%), mitral insufficiency in 21 (7.9%), and PDA in 12 (4.5%). These results suggest that PFO is the most common structural heart abnormality observed in patients undergoing ECG screening and is often accompanied by other anatomical conditions such as ASD, mitral insufficiency, and PDA.

Table 7 also details associations between a positive screening for a prolonged QTc interval, followed by further evaluation, and structural heart abnormalities in 116 patients (representing 0.27‰ of those screened; 5.2% of those enrolled at ECG 1.0). Among these patients, the most frequent findings were 62.1% with a PFO combined with other structural abnormalities, 5.2% with PFO and VSD, and 3.4% with PFO and PDA. Finally, 10.3% of patients had an isolated ASD OS.

## 4. Discussion

This study investigated the usefulness of neonatal ECG screening in diagnosing LQTS [41], identifying heart rhythm abnormalities, and detecting structural heart conditions that might contribute to morbidity and mortality in neonates and infants [43,44].

### 4.1. Electrocardiographic Neonatal Screening

This retrospective study investigated ECG screening for LQTS on 42,200 newborns over 16 years. Males exhibited a significantly lower frequency of LQTS. The study also identified variations in the mean QTc interval over time. Follow-up of these newborns proved valuable in detecting children with LQTS and other heart rhythm abnormalities. Furthermore, neonatal screening for LQTS was beneficial in identifying children with undiagnosed CHDs, significantly reducing the risk of mortality, morbidity, and disability.

The exact causes of SIDS remain unclear. While arrhythmias and cardiovascular changes are suspected to play a role in infant deaths [45], with some studies suggesting a link to prolonged QTc interval in early life [22], definitively establishing a cardiac cause for SIDS is challenging [46]. A significant breakthrough in understanding SIDS came with the triple-risk model. This model successfully integrates epidemiological, physiological, and neuropathological data associated with SIDS, offering a more comprehensive view of the complex pathophysiology behind the syndrome [47].

LQTS can cause abnormal heartbeats and an increased risk of sudden cardiac arrest [36]. Its estimated incidence is around 1 case per 2000–2500 individuals [48]. Although LQTS is rare, it can be dangerous in infants and children as their cardiovascular system is still in the developmental stage. Moreover, an abnormal heart rhythm, such as VT or ventricular arrhythmia, could lead to sudden cardiac arrest [49]. Birth defects of the heart (structural heart abnormalities) raise the chances of experiencing a sudden cardiac arrest later in life [44].

The QTc undergoes a physiological prolongation from the second month of life, returning to typical values at six months. SIDS may be due to a mechanism similar or identical to that responsible for LQTS [23]. The prevalence of LQTS varies from 1 in 20,000 to 1 in 5000 [37]. A prospective study demonstrated that in 50% of SIDS victims, the QTc was >435 ms [42]. The QT interval was increased in SIDS victims (QTc ≥ 440 ms, exceeding the 97.5th percentile). The risk of SIDS in infants with QTc > 435 ms was calculated to be 41 times higher than that of children with a normal QTc [22]. LQTS and SIDS share similar phenotypes, such as prolonged QTc interval, reduced parasympathetic tone, and/or autonomic nervous system imbalance [50].

The prevalence of LQTS identified in children undergoing ECG screening for LQTS is 5.32%. The prevalence of Long QTc was 1 in 2381 (0.42‰), similar to that found in the study by Schwartz et al. (1 in 2534) [37]. A prospective study identifies 1.4‰ out of 685 cases with definite LQTS [51]. However, our data might be underestimated because follow-up data could not be obtained for all children.

### 4.2. Genetic Investigation of LQTS

LQTS is a type of heart condition where tiny channels in the heart muscles, essential for electrical signals, do not work properly. This is thought to be responsible for roughly 12% of SIDS cases [36]. Screening in the neonatal period allows the identification of “at-risk” infants before the peak incidence of SIDS (2–6 months).

ClinGen, the Clinical Genome Resource, is valuable for understanding the genetic basis of LQTS. However, the Long QT Syndrome Gene Curation Expert Panel (GCEP) was formed in 2019, focusing on 17 genes associated with SQTS (Short QT Syndrome). Their evaluation of these genes was completed in 2020 (https://www.clinicalgenome.org/affiliation/40025/, accessed on 10 February 2024).

Between 2001 and 2017, we identified 11 children carrying pathogenic mutations of LQTS: six *LQT1* (*KCNQ1*; 54.5%), three *LQT2* (*KCNH2*; 27.3%), and one *LQT3* (*SCN5A*; 9.1%). One case (9.1%) presented a combined mutation of *LQT1* (*KCNH2*) and *LQT3* (*SCN5A*). It is estimated that around 10% of SIDS cases may be caused by LQTS [23]. Follow-up evaluation predominantly shows patients to be asymptomatic, except for one case with KCNQ1 mutation that manifested a syncopal episode, highlighting the importance of genotype–phenotype correlation. For the remaining children in our case series, the clinical diagnosis of LQTS was confirmed, but the genetic mutation was not identified.

Mutations in the *LQT1* gene (*KCNQ1*) cause defects in potassium channels, leading to the LQT1 phenotype, the most common form of LQTS. Carriers of *LQT1* have a higher risk of ventricular arrhythmias and fatal events [52]. Our findings align with previous reports [37,53], demonstrating a higher prevalence of LQT1 compared to other subtypes within the LQTS population studied. Genetic screening has allowed the identification and genetic characterization of affected family members with the syndrome. The nucleotide substitution in the *LQT3* gene (*SCN5A*; *c.647C>T*) has been described in patients with LQTS, Brugada syndrome, atrial fibrillation, and SIDS [54,55]. *LQT3* patients exhibited a more aggressive course, with a high rate (20%) of life-threatening cardiac events. In contrast, with one exception, both *LQT1* and *LQT2* patients had uneventful clinical courses, and outcomes, with no deaths and only one heart transplant [56].

After discovering the first three LQTS-susceptibility genes (*KCNQ1*, *KCNH2*, and *SCN5A*), a total of 17 genes have been identified, with seven genes discovered by Ackerman et al. [25]. However, over half of the genes reported as responsible for LQTS have limited or contested evidence supporting their causality in the disease [57].

LQTS typically follows an autosomal dominant inheritance pattern, with most cases inherited from an affected parent. De novo pathogenic variants, where the mutation arises spontaneously, contribute minimally to LQTS. The penetrance of the disorder can vary [58]. The study has demonstrated the potential of neonatal screening to identify families at risk of LQTS. Therefore, the benefit of neonatal screening has been extended to the relatives of children with LQTS. Neonatal ECG screening reveals variations in the length of the QTc interval, suggesting the need for comprehensive assessments even in the absence of specific genetic mutations.

The therapeutic management outlined in the children’s protocol periodizes early identification and personalized treatment. Beta-blockers, such as propranolol, nadolol, and metoprolol, form the cornerstone of therapy, with dosages tailored to individual needs. This personalized approach underscores the importance of individualized management in Long QT.

### 4.3. Single and Multiple ECG Abnormalities

Neonatal ECG screening identified cardiac rhythm anomalies in a small percentage of screened neonates, including right bundle branch block (2.33%), left axis deviation (0.39%), and nonspecific ventricular repolarization anomalies (0.30%). The most frequent combined ECG anomalies were the right bundle branch block with right ventricular prevalence (0.14%) and the right bundle branch block with left axis deviation (0.06%). As reported in the literature, echocardiographic assessment in these patients with prolonged QTc and/or other ECG abnormalities helped identify CHDs [37].

### 4.4. Congenital Heart Diseases and/or Valvulopathies

Routine ECG screening offers a valuable tool for identifying unrecognized CHDs that may go undetected by prenatal ultrasound and standard neonatal examinations in asymptomatic patients [44]. A study found that postnatal echocardiographic screening, while sometimes recommended, does not significantly improve the detection rate of critical or severe heart defects in newborns without prenatal diagnosis or clinical signs. This approach also carries a high rate of false positives, leading to unnecessary follow-up procedures [59].

Neonatal ECG screening identified structural heart abnormalities in 6.3‰ of screened neonates. Patent foramen ovale (PFO) was the most prevalent anomaly, detected either alone (3.9‰) or with other heart defects (2.4‰). Mitral valve insufficiency was less common (0.28‰).

Overall, the frequency of cardiac anomalies identified in children enrolled for serial neonatal ECG screening for suspected prolonged QTc was 5.57%, with PFO, either single (3.21%) or associated with other structural heart anomalies, being the most common (4.01%). Subsequently, isolated ASD OS (5.3‰) or associated with other cardiac anomalies was less commonly observed (6.7‰). Echocardiographic evaluation in these patients with prolonged QTc and/or other ECG abnormalities facilitated the identification of CHDs, as reported in the literature [37].

It has been reported that the overall incidence of CHDs is approximately 6–9‰ [60]. However, neonatal clinical examination generally cannot detect all forms of CHD [61]. The incidence of CHDs in asymptomatic neonates born at high altitudes was 27.8%, with ASD OS (62.7%), PDA (21.9%), VSD (2.9%), and multiple defects (12.6%) being observed [62]. Almost all CHD cases were simple forms with left-to-right shunting, including ASD OS, PDA, and VSD [62].

### 4.5. Summary

Neonatal ECG screening aims to identify heart abnormalities that increase the risk of cardiovascular complications and death. In this study, 42,200 newborns underwent screening. Among them, 2245 (5.3%) required further evaluation with serial ECGs due to concerns about a prolonged QTc interval. Ultimately, 18 children were diagnosed with prolonged QTc, and 11 of them had an identified genetic mutation causing LQTS. Propranolol was the primary medication used to treat prolonged QT.

An additional benefit of routine neonatal ECG screening is the detection of other arrhythmogenic or structural cardiovascular abnormalities that prenatal and standard newborn examinations might have missed. Early diagnosis in asymptomatic infants allows for prompt medical or surgical therapy, potentially reducing future health problems and death.

## 5. Conclusions

This study highlights the practical value of neonatal ECG screening between 20 and 40 days of life. The screening effectively diagnosed LQTS and other cardiac rhythm anomalies in newborns, even raising suspicion of structural heart pathologies early. Notably, genetic mutations, particularly channelopathies, were identified in over half of the children with prolonged QT. This opens the door for early interventions like beta-blocker treatment. The cost–benefit analysis shows that screening saves newborns with LQTS and offers additional benefits. These include mutations and/or prolonged QTc in family members. Furthermore, detecting other ECG abnormalities and CHDs further expands the pool of children who can benefit from this screening.

## Figures and Tables

**Table 1:** The table shows the percentage of children (with the percentage of males in brackets) for each QTc ≥ cut-off value (listed in the first column). These percentages are based on ECGs taken at regular intervals (ECG 1.0 to ECG 4.0 and ECG 1.1 to ECG 1.4) and Holter ECGs (shown in the last column). To aid interpretation, the color of each box corresponds to the percentage of children (with the percentage of males in brackets) who have a QTc exceeding the specified cut-off and falling within the range indicated in the table legend.

Included at Follow-Up, QTc (ms)	ECG 1.0, n. (% Males)		ECG 2.0, n. (% Males)	ECG 3.0, n. (% Males)		ECG 4.0, n. (% Males)		ECG 1.1, n. (% Males)	ECG 2.1, n. (% Males)		ECG 3.1, n. (% Males)	ECG 4.1, n. (% Males)		Holter 24 h ECG, n. (% Males)
Infant enrolled	2245 (39.9)		667 (35.2)	254 (29.1)		52 (25)		164 (37.8)	119 (32.8)		87 (24.1)	23 (26.1)		44 (31.8)
	% (% males)		% (% males)	% (% males)		% (% males)		% (% males)	% (% males)		% (% males)	% (% males)		% (% males)
≥435	98.8 (38.9)		99.3 (35.2)	95.7 (58.9)		55.8 (24.1)		99.4 (37.4)	72.6 (32.8)		100 (24.1)	100 (26.1)		100 (30.2)
≥440	86.8 (39.8)		88.0 (34.2)	89.4 (27.3)		92.3 (27.1)		89.0 (37.0)	64.6 (32.1)		92.0 (23.8)	91.3 (28.6)		95.5 (25.6)
≥445	37.8 (37.6)		46.0 (27.4)	59.8 (24.3)		57.7 (13.3)		53.0 (32.2)	40.2 (24.2)		62.1 (22.2)	60.9 (14.3)		95.5 (31.0)
≥450	33.4 (37.8)		42.3 (26.2)	55.9 (25.4)		53.8 (14.3)		48.2 (32.9)	38.4 (25.4)		58.6 (23.5)	56.2 (14.4)		86.4 (23.2)
≥455	14.7 (34.5)		22.8 (22.4)	26.8 (26.5)		34.6 (11.1)		26.8 (29.5)	26.2 (23.3)		26.4 (26.1)	39.1 (11.1)		68.2 (26.7)
≥460	9.6 (30.6)		13.2 (14.8)	17.3 (29.5)		19.2 (20.0)		20.1 (30.3)	16.5 (18.4)		14.9 (30.8)	21.7 (20)		59.1 (15.9)
≥465	4.9 (33.3)		8.6 (8.3)	8.7 (18.2)		11.5 (33.3)		12.8 (42.9)	7.3 (16.7)		4.6 (0)	13.0 (33.3)		45.5 (25.0)
≥470	3.6 (34.6)		5.4 (5.6)	5.4 (0)		3.8 (0)		7.9 (38.5)	7.3 (16.7)		4.6 (0)	4.3 (0)		29.5 (7.9)
≥475	0.67 (26.7)		2.4 (6.3)	3.5 (0)		3.8 (0)		2.4 (25)	3.7 (16.7)		3.4 (0)	4.3 (0)		22.7 (20.0)
≥480	0.49 (36.4)		2.4 (6.3)	3.5 (0)		3.8 (0)		1.8 (33.3)	3.7 (16.7)		3.4 (0)	4.3 (0)		20.5 (5.5)
≥485	0.22 (20)		2.1 (7.1)	2.0 (0)		-		0.61 (0)	3.0 (20)		1.1 (0)	-		18.2 (25.0)
≥490	0.22 (20)		1.6 (9.1)	2.5 (0)		-		0.61 (0)	2.4 (25)		1.1 (0)	-		15.9 (4.3)
≥495	0.22 (0)		1.3 (0)	1.2 (0)		-		0.61 (0)	1.8 (0)		1.1 (0)	-		11.4 (20.0)
≥500	0.22 (0)		1.3 (0)	1.2 (0)		-		-	-		-	-		9.1 (2.4)
≥510	0.22 (0)		0.9 (0)	1.2 (0)		-		-	-		-	-		4.5 (1.2)
From ECG 1.0 to ECG 4.0		100–80%			80–60%		60–40%			40–20%			20–0%	
From ECG 1.1 to ECG 4.1		100–80%			80–60%		60–40%			40–20%			20–0%	
Holter 24 h ECG		100–80%			80–60%		60–40%			40–20%			20–0%	

Legend: ECG, electrocardiogram.

**Table 2:** The table displays the mean QTc values ± SD of infants whose QTc values are greater than or equal to (≥) a predefined cut-off value listed in the first column. The values are presented for consecutive ECGs (from ECG 1.0 to ECG 4.0 and from ECG 1.1 to ECG 4.1). The last column shows the mean QTc values ± SD from a Holter ECG for infants meeting the same criteria (predefined cut-off value in the first column). For improved clarity, the color of each box corresponds to the range of mean QTc values ± SD that fall within 0–20% of the total children included at each consecutive ECG and Holter ECG measurement.

Included at Follow-Up (QTc, ms)	ECG 1.0, Mean QTc ± SD (Min–Max) ms	ECG 2.0, Mean QTc ± SD (Min–Max) ms	ECG 3.0, Mean QTc ± SD (Min–Max) ms	ECG 4.0, Mean QTc ± SD (Min–Max) ms	ECG 1.1, Mean QTc ± SD (Min–Max) ms	ECG 2.1, Mean QTc ± SD (Min–Max) ms	ECG 3.1, Mean QTc ± SD (Min–Max) ms	ECG 4.1, Mean QTc ± SD (Min–Max) ms	Holter 24 h ECG, Mean QTc ± SD (Min–Max) ms
Infant enrolled	445.9 ± 9.4 (392–513)	448.1 ± 12.1 (427–513)	450.2 ± 12.7 (428–520)	450.5 ± 11.2 (435–482)	449.6 ± 11.9 (433–513)	451.7 ± 14.8 (435–513)	450.4 ± 12.1 (435–520)	451.3 ± 11.6 (435–482)	465.7 ± 19.2 (430–511)
≥435	446.1 ± 9.2	448.3 ± 12.1	450.3 ± 12.6	449.9 ± 10.9	449.7 ± 11.9	451.7 ± 14.8	450.4 ± 12.1	451.3 ± 11.6	466.5 ± 18.6
≥440	447.5 ± 9.0	449.8 ± 11.9	452.0 ± 12.2	451.8 ± 10.6	451.3 ± 11.5	453.6 ± 14.6	451.7 ± 11.7	452.9 ± 11.0	467.1 ± 18.3
≥445	455.2 ± 8.7	457.5 ± 12.1	457.0 ± 12.0	458.0 ± 8.7	457.9 ± 10.7	460.9 ± 14.1	456.3 ± 11.7	458.5 ± 9.0	467.2 ± 18.3
≥450	456.3 ± 8.6	458.4 ± 12.2)	457.7 ± 12.2	458.8 ± 8.5	459.0 ± 10.6	461.5 ± 14.1	456.9 ± 11.8	459.4 ± 8.7	469.4 ± 17.8
≥455	463.7 ± 8.3	465.2 ± 13.3	465.6 ± 13.7	463.1 ± 7.6	465.8 ± 9.8	466.7 ± 14.4	464.7 ± 14.1	463.1 ± 7.8	474.1 ± 17.2
≥460	467.0 ± 8.6	470.8 ± 15.1	470 ± 15.4	457.2 ± 8.2	468.5 ± 10.0	472.5 ± 15.7	470.4 ± 16.9	467.2 ± 8.7	476.9 ± 16.7
≥465	472.6 ± 8.9	479.2 ± 16.2	479.3 ± 17.4	471.3 ± 8.3	472.7 ± 10.3	484.7 ± 16.5	489.5 ± 20.7	(466–482)	481.6 ± 16.4
≥470	475.0 ± 9.3	483.7 ± 16.4	488.5 ± 17.4	482	476.9 ± 11.3	484.7 ± 16.5	489.5 ± 20.7	482	489.9 ± 14.4
≥475	488.4 ± 15.7	499.1 ± 13.3	495.4 ± 16.7	482	487.3 ± 17.3	497.5 ± 14.2	(482–520)	482	495.1 ± 12.2
≥480	492.9 ± 16.2	499.1 ± 13.3	495.4 ± 16.7	482	513	497.5 ± 14.2	520	482	497.3 ± 10.5
≥485	508.4 ± 10.3	501 ± 11.8	506 ± 15.2	-	513	501 ± 12.7	520	-	499.5 ± 8.8
≥490	508.4 ± 10.3	506.4 ± 8.6	506 ± 15.2	-	513	505 ± 10.4	520	-	501.6 ± 7.1
≥495	513	510 ± 3.1	(511–520)	-	513	(506–513)	520	-	504.6 ± 5.9
≥500	513	510 ± 3.1	(511–520)	-	-	-	-	-	506.0 ± 5.8
≥510	513	512.0 ± 1.1	(511–520)	-	-	-	-	-	511
From ECG 1.0 to ECG 4.0					20–0%				
From ECG 1.1 to ECG 4.1					20–0%				
Holter 24 h ECG					20–0%				

Legend: ECG, electrocardiogram; SD, standard deviation.

**Table 3:** Table displays the mean QTc values and SD (obtained from ECGs 1.1 to 4.1 and Holter ECG) for children undergoing further diagnostic investigation.

	*N*	Mean ± SD	Minimum–Maximum
ECG 1.1 QTc ms	27	452.5 ± 13.1	435–476
ECG 2.1 QTc ms	25	458.5 ± 19.1	435–511
ECG 3,1 QTc ms	21	462.2 ± 20.9	441–520
ECG 4.1 QTc ms	12	457.0 ± 11.2	442–482
ECG Holter, QTc ms	18	452.9 ± 26.2	417–538

Legend: ECG, electrocardiogram; SD, standard deviation.

**Table 4:** Results of QTc and therapy undertaken by seven children with Long QTc in whom the effects of genetic investigations were adverse.

SEX	QTc msc (ECG)	QTc ms (ECG Holter)	Therapy	Dosage
F	468	460	Propranolol	2 mg/kg, bid
F	402	441	Propranolol	2 mg/kg, qd
F	413	449	Propranolol	2 mg/kg, tid
M	433	440	Propranolol	2 mg/kg, tid
F	494	537	Nadolol	¾ cp (40 mg), tid
F	EXTENDED	EXTENDED	Metoprolol	8 mg, bid
F	EXTENDED	EXTENDED	Metoprolol	8 mg, bid

Legend: bid, twice a day; ECG, electrocardiogram; qd, once a day; tid, three times a day.

**Table 5:** Mutations were identified and therapy was initiated in 11 children, including 2 siblings, affected by Long QTc in the screening ECG and Holter ECG.

SEX	MUTATION	Familiarity	QTc ms (ECG)	QTc ms (ECG Holter)	Therapy	Dosage
F	*SCN5A* (*LQT3*; *c.647C>T*)	No	427	438	Propranolol	2 mg/kg, tid
F	*KCNH2* (*LQT2*)	Father	458	461	Propranolol	20 mg
F	*KCNH2* (*LQT2*; *c.1196C>T*)	No	438 and short PR	-	-	-
F	*KCNH2* (*LQT2*; *c.3367G>C*)	No	452	462	Propranolol	3 mg/kg, tid
F	*KCNH2* (*LQT2*; *c.2560T>G*), *SCN5A* (*LQT3*; *c.5845G>A*)	No	-	-	-	-
F	*KCNQ1* (polymorphism *SCN5A-H558R*, *KCNH2*-*K897K*, e *KCNE1*-*S38G*)	No	448	465	Propranolol	2 mg/kg, tid
F	*KCNQ1* (*LQT1*)	Mother	NA	NA	Nadolol	1.5 mg/Kg/day
F	*KCNQ1* (*LQT1*)	Mother and maternal grandfather	NA	NA	-	-
M	*KCNQ1* (*LQT1*)	Mother and sister	434	465	Propranolol	¾ + ½ + ½
M *	*KCNQ1* (*LQT1*)	Father (asymptomatic, QTc in the norm)	NA	NA	Nadolol	1 mg/Kg, qd
M *	*KCNQ1* (*LQT1*)	Father (asymptomatic, QTc in the norm)	NA	NA	Nadolol	1 mg/Kg, qd

* brothers. Legend: qd, once a day; tid, three times a day; NA, not available.

**Table 6:** ECG abnormalities, different from Long QTc, were detected in subjects identified by neonatal ECG screening.

Single ECG Abnormality	Patients, No. (% of Total)	Multiple ECG Abnormality	Patients, No. (% of Total)
Right bundle branch focal block	983 (54.5)	Right bundle branch focal block + Right ventricular prevalence	61 (30.8)
Left axial deviation	166 (9.2)	Right bundle branch focal block + Left axial deviation	26 (13.1)
Nonspecific abnormalities of ventricular repolarization	128 (7.1)	Right bundle branch focal block + Nonspecific alterations of repolarization	8 (4.0)
Ventricular extrasystole	83 (4.6)	Right bundle branch focal block + Supraventricular extrasystole	6 (3.0)
Supraventricular extrasystole	82 (4.5)	Right bundle branch focal block + Ventricular extrasystole	6 (3.0)
Complete right bundle branch block	66 (3.7)	Right bundle branch focal block + High voltages of QRS	5 (2.5)
High-voltage QRS	46 (2.5)	Right bundle branch focal block + PQ at upper limits	5 (2.5)
Ventricular pre-excitation	32 (1.8)	Right bundle branch focal block + Negative T wave	2
Increased P wave amplitude	28 (1.5)	Right bundle branch focal block + Inf Q waves	2
FP at upper limits	27 (1.5)	Right bundle branch focal block + Right ventricular head + Left axial deviation	2
Low QRS voltages	20 (1.1)	Right bundle branch focal block + Sinus tachycardia	2
Tachycardia sinusale	20 (1.1)	Blocco focale di branca dx + Deviazione assiale sx + Extrasistolia sopraventricolare	1
Bradicardia sinusale relativa	18 (1.0)	Right bundle branch focal block + Supraventricular extrasystole + Ventricular extrasystole	1
FP at lower limits	16 (0.9)	Right bundle branch focal block + Ventricular parasystole	1
AV conduction at the upper limits	10 (0.6)	Right bundle branch focal block + Right ventricular prevalence + PQ at lower limits	1
Positive T	10 (0.6)	Right branch focal block + Migrant steplight	1
Ectopic atrial rhythm	9 (0.5)	Right bundle branch focal block + Right ventricular and atrial prevalence	1
Positive T wave	8 0.4)	Right bundle branch focal block + Right ventricular prevalence + PQ at upper limits	1
Migrant step marker	8 (0.4)	TOTAL Right bundle branch focal block	132 (66.7)
Right axial deviation	7 (0.4)	Right ventricular head + left axial deviation	17 (8.6)
Left front hemiblock	5 (0.3)	Right ventricular head + Deep Q waves	7 (3.5)
Delayed right intraventricular conduction	4 (0.2)	Right ventricular prevalence + PR at the experimental limits	6 (3.0)
Respiratory sinus arrhythmia	3 (0.2)	Right ventricular head + High QRS voltages	4 (2.0)
ST elevation	3 (0.2)	Right ventricular prevalence + Nonspecific abnormalities of ventricular repolarization	4 (2.0)
Negative T	3 (0.2)	Right ventricular prevalence + Ventricular extrasystole	4 (2.0)
Dextrocardia	2 (0.1)	Right ventricular head + Low QRS voltages	4 (2.0)
QTc and PQ at the lower limits	2 (0.1)	Right ventricular prevalence + Nonspecific abnormalities of ventricular repolarization	3 (1.5)
Ventricular hyperkinetic arrhythmia	1	Right ventricular prevalence + Supraventricular extrasystole	3 (1.5)
Appearance S1-Q3	1	Right ventricular prevalence + PQ at upper limits	2 (1.0)
AV dissociation	1	Right ventricular head + Appearance S1-Q3	1
Right atrial engagement	1	Right ventricular prevalence + AV conduction at upper limits	1
Increased amplitude P waves	1	TOTAL Right ventricular prevalence	56 (28.3)
Septal Q waves	1	Supraventricular extrasystole + Ventricular extrasystole	7 (3.5)
Flat T waves	1	Ventricular pre-excitation + Right bundle branch focal block	2 (1.0)
AV conduction extension	1	Ventricular pre-excitation + Right ventricular head	2 (1.0)
qR in inferolateral site	1	TOTAL Ventricular pre-excitation	4 (2.0)
Biventricular overloads	1	Left axial deviation + Sinus tachycardia	1
Signs of bi-atrial engagement	1	Left front hemiblock + Left axial deviation	1
Right overload	1	Biventricular hypertrophy + Supraventricular extrasystole	1
Right ventricular overload	1	PQ at lower limits + Supraventricular extrasystole	1
Diphasic T	2 (0.1)	FP at lower limits + Probable junctional rhythm in migrant stepper	1
Paroxysmal supraventricular tachycardia	1	Ventricular pre-excitation + Supraventricular extrasystole	1
Total	1805	Migrant steplight + Supraventricular extrasystole	1
		TOTAL	198

Congenital heart diseases and/or valvulopathies.

**Table 7:** This table shows the prevalence of congenital heart diseases (CHDs) and/or valvulopathies in children, categorized by the presence of ECG abnormalities (left columns) and a prolonged QTc interval (right columns).

Structural Alteration of the Heart Associated with ECG Abnormalities	Patients, n. (%)	Structural Alterations of the Heart Associated with Long QTc	Patients, n. (%)
PFO	164 (61.4)	PFO	72 (62.1)
PFO/IAD OS	25 (9.4)	PFO + IVD	6 (5.2)
PFO + Mitral insufficiency	21 (7.9)	PFO + PDA	4 (3.4)
PFO + PDA	12 (4.5)	PFO/IAD OS	3 (2.4)
PFO + IVD	6 (2.2)	PFO + Mitral insufficiency	3 (2.4)
PFO/IAD OS + Mitral insufficiency	3 (1.1)	PFO + Aortic insufficiency	1
PFO + PDA + Mitral insufficiency	3 (1.1)	PFO + Tricuspid insufficiency	1
PFO + Aortic insufficiency	2	IAD OS	12 (10.3)
PFO/IAD OS + IVD	1	IAD OS + IM	1
PFO/IAD OS + PDA	1	IAD OS + Tricuspid insufficiency	1
PFO + Aortic coarctation + Mitral insufficiency	1	IAD OS + Aortic dysplasia	1
PFO + Tricuspid insufficiency	1	PDA	4 (3.2)
PFO + Flow acceleration at the level of the aortic isthmus without obstructive gradient + Mitral insufficiency	2	IVD	3 (2.4)
Mitral insufficiency	12 (4.5)	Mitral insufficiency	2
IAD OS	2	Aortic Insufficiency	1
IAD OS + Tricuspid insufficiency	2	Pulmonary stenosis	1
PDA	2	TOTAL	116
PDA + Mitral insufficiency	2		
IVD	1		
Pulmonary insufficiency	1		
Tricuspid insufficiency	1		
Mild aortic insufficiency in apparently tricuspid valve	1		
Multiple ventricular echo-dense neoformations, referable in the first hypothesis to rhabdomyoma	1		
TOTAL	267		

Legend: IAD, interatrial defect; IVD, interventricular defect; OS, ostium secundum; PFO, patent foramen ovale; PDA, patent ductus arteriosus.

## Data Availability

The original contributions presented in the study are included in the article.
